# Supplementary material for: Adult and Elderly Risk Factors of Mortality in 23,614 Emergently Admitted Patients with Rectal or Rectosigmoid Junction Malignancy
Source: Int J Environ Res Public Health. 2022 Jul 27;19(15):9203. doi: 10.3390/ijerph19159203 (PMC9368534; doi:10.3390/ijerph19159203)
Supplement: Supplementary file 1 [file ijerph-19-09203-s001.zip › ijerph-1819663-supplementary.pdf]

International Journal of
Environmental Research and Public Health

MDPI

Supplementary Table S1. Secondary diagnoses of patients emergently admitted with a primary diagnosis of malignant neoplasm of rectum or rectosigmoid junction (NIS 2004-2014). Data was stratified according to survival status.

Comorbidities and Secondary Diagnoses (ICD-9 Codes)	Adult, N (%)		P value	Elderly, N (%)		P value
	Survived	Deceased		Survived	Deceased	
Observations	12,594 (95)	624 (5)		15,082 (94)	1,006 (6)	
Tuberculosis (010.0-018.96)	3 (0.0)	0 (0)	0.700	2 (0.0)	0 (0)	0.720
Bacterial Infections Other than Tuberculosis (020.0-041.9, 790.7)	1,007 (8)	157 (25)	<0.001	1,450 (10)	277 (28)	<0.001
Nonbacterial Infections (042, 795.71, V08, 045.0-139.8, 790.8, and/or presence of Comorbidity of AIDS)	838 (7)	59 (10)	0.007	588 (4)	51 (5)	0.070
Diabetes (250.0-250.93, V58.67, &/or presence of Comorbidity of Diabetes Uncomplicated or Diabetes Chronic Complications)	1,890 (15)	66 (11)	0.002	3,432 (23)	154 (15)	<0.001
Hypertension (401.0-405.99, 796.2, &/or presence of Comorbidity of Hypertension)	4,396 (35)	155 (25)	<0.001	8,842 (59)	442 (44)	<0.001
Anemia &/or Hemorrhage (280.0-285.9, 784.7, 784.8, &/or presence of Comorbidity of Anemia)	5,785 (46)	242 (39)	<0.001	8,132 (54)	417 (42)	<0.001
Respiratory Diseases (415.0-417.9, 460-519.9, 784.91, 786, &/or presence of Comorbidity of COPD, ILD or Pulmonary Circulation Disease)	2,737 (22)	288 (46)	<0.001	5,129 (34)	560 (56)	<0.001
Coagulopathy (286.0-286.9, 790.92, V58.61, V58.63, &/or presence of Comorbidity of Coagulopathy)	745 (6)	111 (18)	<0.001	1,423 (9)	147 (15)	<0.001
Cardiac Diseases (391.X, 392.0, 393.398.99, 410.0-414.9, 420.0-429.9, 794.3X, 785.XX, &/or presence of Comorbidity of CHF or Valvular Diseases)	2,374 (19)	215 (35)	<0.001	6,949 (46)	568 (57)	<0.001
Cerebrovascular Diseases (325, 430-438)	144 (1)	4 (1)	0.240	666 (4)	53 (5)	0.210
Peripheral Vascular Diseases (440-457.9, &/or presence of Comorbidity of Peripheral Vascular Disorders)	1,455 (12)	65 (10)	0.390	2,546 (17)	175 (17)	0.670
Liver Diseases (570-573.9, 790.4, 794.8, &/or presence of Comorbidity of Liver Diseases)	799 (6)	95 (15)	<0.001	542 (4)	73 (7)	<0.001
Diseases of Digestive System other than Liver (530.00-569.9, 574.0-579.9, 787, 001.0-009.3, &/or presence of Comorbidity of Peptic Ulcer)	8,248 (66)	369 (59)	0.001	10.736 (71)	674 (67)	0.005
Diseases of Oral Cavity, Salivary Glands, and Jaws (520-529)	74 (1)	8 (1)	0.031	61 (0.4)	12 (1)	<0.001
Nutritional/Weight Disorders (260-273.9, 275.XX,277.0-278.8, 783.XX, 799.3-799.4, &/or presence of Comorbidity of Weight Loss)	4,488 (36)	278 (45)	<0.001	6,787 (45)	451 (45)	0.920
Endocrine Diseases (240.0-259.9, 991.0-992.9, &/or presence of Comorbidity of Endocrine Diseases)	2,418 (19)	89 (14)	0.002	4,709 (31)	211 (21)	<0.001
Genitourinary System Diseases (580.0-629.9, 403.XX, 791.XX, 788.XX, &/or presence of Comorbidity of Renal Diseases)	3,905 (31)	277 (44)	<0.001	6,395 (42)	544 (54)	<0.001
Neurological Diseases (317.0-326, 330.0-337.9, 340-359.9, 392, 780.0-780.09, 780.2-780.4, 317-319, 290.XX, 294.XX, 781.0-782.0, &/or presence of Comorbidity of Paralysis or Other Neurological Disorders or Paralysis)	1,160 (9)	113 (18)	<0.001	2,940 (20)	233 (23)	0.005
Diseases of the Musculoskeletal System and Connective Tissue (274.XX, 710.0-739, &/or presence of Comorbidity of Rheumatoid Arthritis or Lupus)	1,474 (12)	46 (7)	<0.001	3,138 (21)	140 (14)	<0.001
Fluid and Electrolyte Disorders (275.0-276.9, 458.0-459.9, &/or presence of Comorbidity of Fluid and Electrolyte Disorders)	4,029 (32)	361 (58)	<0.001	5,892 (39)	587 (58)	<0.001
Neoplasms (140.0-239.9, V10.XX, &/or presence of Comorbidity of Lymphoma, Metastatic Diseases, or Tumor)	7,580 (60)	510 (82)	<0.001	8,460 (56)	623 (62)	<0.001
Platelet and White Blood Cell Diseases (204.0-208.92, 287.0-288.9, 238.71)	1,004 (8)	80 (13)	<0.001	1,056 (7)	118 (12)	<0.001
Psychiatric Diseases (293.XX, 295.0-302.9, 306.0-316, 780.1, V62.8, V15.4, &/or presence of Comorbidity of Psychoses)	1,978 (16)	79 (13)	0.040	1,999 (13)	133 (13)	0.980
Skin Diseases (680.0-709.9, 782.1-782.9)	876 (7)	91 (15)	<0.001	1,228 (8)	98 (10)	0.070

Trauma, Burns and Poisoning (800-999)	1,922 (15)	181 (29)	<0.001	2,639 (18)	329 (33)	<0.001
Drug Abuse/Withdrawal/Dependence (292.0-292.9, 304.0-304.93, 305.2-305.93, &/or presence of Comorbidity of Drug Abuse)	440 (4)	11 (2)	0.020	122 (1)	8 (1)	0.960
Alcohol Abuse/Withdrawal/Dependence (291.0-291.9, 303.0-303.93, 305.0-305.03, &/or presence of Comorbidity of Alcohol Abuse)	745 (6)	23 (4)	0.020	387 (3)	17 (2)	0.090
Tobacco Use (305.1)	3,504 (28)	95 (15)	<0.001	2,817 (19)	108 (11)	<0.001
Long-Term Medications/Radiotherapy (V58.0-V58-2, V58.62, V58.64-V58.66, V58.68-V58.69)	676 (5)	26 (4)	0.190	945 (6)	39 (4)	0.002
Social Factors (V60.0-V62.6, V63.0-V64.3, V15.81)	498 (4)	13 (2)	0.018	439 (3)	16 (2)	0.014
Sleep Disorders (327, 780.5, V69.4, V69.5)	348 (3)	5 (1)	0.003	330 (2)	15 (2)	0.140
Lack of Physical Exercise (V69.0)	0 (0)	0 (0)		3 (0.0)	0 (0)	0.660
Inappropriate Diet and Eating Habits (V69.1)	0 (0)	0 (0)		0 (0)	0 (0)	
High Risk Lifestyle Behaviors (V69.2, V69.3)	0 (0)	0 (0)		0 (0)	0 (0)	
Body Mass Index of Less than 18.9 (V85.0)	236 (25)	16 (43)		272 (32)	24 (62)	
Body Mass Index of 19-24.9 (V85.1)	257 (27)	8 (22)	0.090	256 (30)	12 (31)	<0.001
Body Mass Index of 25.0-29.9 (V85.21-V85.25)	133 (14)	5 (14)		116 (14)	2 (5)	
Body Mass Index of 30.0 and over (V85.30-V85.45)	313 (33)	8 (22)		209 (25)	1 (3)	
